# Late effects of high-dose methotrexate treatment in childhood cancer survivors—a systematic review

**DOI:** 10.1186/s12885-021-09145-0

**Published:** 2022-03-14

**Authors:** Eveline Daetwyler, Mario Bargetzi, Maria Otth, Katrin Scheinemann

**Affiliations:** 1 Department of Medical Oncology, University Hospital Basel, Basel, Switzerland; 2 Faculty of Medicine, University of Basel, Basel, Switzerland; 3 Division of Hematology/Oncology, University Medical Clinic, Kantonsspital Aarau, Aarau, Switzerland; 4 Division of Hematology-Oncology, Department of Pediatrics, Kantonsspital Aarau AG, Tellstrasse 25, CH-5001 Aarau, Switzerland; 5 Department of Oncology, Hematology, Immunology, Stem Cell Transplantation and Somatic Gene Therapy, University Children’s Hospital Zurich - Eleonore Foundation, Zurich, Switzerland; 6 Department of Pediatrics, McMaster Children’s Hospital and McMaster University, Hamilton, Canada

**Keywords:** Paediatric cancer, Cancer survivor, High-dose methotrexate, Late effects, Follow-up care, Systematic review

## Abstract

**Background:**

High-dose methotrexate (HD-MTX) is used in the treatment of different childhood cancers, including leukemia, the most common cancer type and is commonly defined as an intravenous dose of at least 1 g/m^2^ body surface area per application. A systematic review on late effects on different organs due to HD-MTX is lacking.

**Method:**

We conducted a systematic literature search in PubMed, including studies published in English or German between 1985 and 2020. The population of each study had to consist of at least 75% childhood cancer survivors (CCSs) who had completed the cancer treatment at least twelve months before late effects were assessed and who had received HD-MTX. The literature search was not restricted to specific cancer diagnosis or organ systems at risk for late effects. We excluded case reports, case series, commentaries, editorial letters, poster abstracts, narrative reviews and studies only reporting prevalence of late effects. We followed PRISMA guidelines, assessed the quality of the eligible studies according to GRADE criteria and registered the protocol on PROSPERO (ID: CRD42020212262).

**Results:**

We included 15 out of 1731 identified studies. Most studies included CCSs diagnosed with acute lymphoblastic leukemia (*n* = 12). The included studies investigated late effects of HD-MTX on central nervous system (*n* = 10), renal (*n* = 2) and bone health (*n* = 3). Nine studies showed adverse outcomes in neuropsychological testing in exposed compared to non-exposed CCSs, healthy controls or reference values. No study revealed lower bone density or worse renal function in exposed CCSs. As a limitation, the overall quality of the studies per organ system was low to very low, mainly due to selection bias, missing adjustment for important confounders and low precision.

**Conclusions:**

CCSs treated with HD-MTX might benefit from neuropsychological testing, to intervene early in case of abnormal results. Methodological shortcomings and heterogeneity of the tests used made it impossible to determine the most appropriate test. Based on the few studies on renal function and bone health, regular screening for dysfunction seems not to be justified. Only screening for neurocognitive late effects is warranted in CCSs treated with HD-MTX.

**Supplementary Information:**

The online version contains supplementary material available at 10.1186/s12885-021-09145-0.

## Introduction

Improvements in diagnosis and treatment of children with cancer have resulted in high survival rates and a growing population of childhood cancer survivors (CCSs) [1, 2]. Amongst other factors, this high survival rate is achieved through intensive treatments, which can cause late effects in potentially every organ system [3, 4].The early detection and treatment of late effects have become a focus of research. Concomitantly, different national long-term follow-up (LTFU) care guidelines have been established to address the need to detect late effects early, recommend treatment and improve the quality of life of CCS. These LTFU care guidelines include those from the Children’s Oncology Group (COG) in the US [5], the United Kingdom Children’s Cancer Study Group Late Effects Group (UKCCLG) [6] and the Dutch Childhood Oncology Group (DCOG) [7]. These guidelines recommend screening for neurocognitive function, bone mineral density, kidney and liver function in exposed CCSs, but the recommendations are not uniform (Supplemental S1).

HD-MTX has proven to be successful in the treatment of a variety of childhood cancers, including acute lymphoblastic leukemia (ALL), non-Hodgkin lymphoma, osteosarcoma and certain high-grade tumours of the central nervous system (CNS) [8–11]. There is no official cut-off dose to define HD-MTX. Ackland et al. defined MTX doses of at least 1 g per body surface area (≥ 1 g/m^2^) given intravenously as high-dose, as from this dose onwards leucovorin rescue is needed [12]. As clinical guidelines use the same cut-off dose, we used it for this systematic review[13]. Despite the successful use of HD-MTX, no systematic review exists of its potential to cause late effects. This systematic review aims to close this gap and to systematically search for evidence of late effects associated with HD-MTX treatment.

## Methods

### Literature search

We conducted the systematic literature search in PubMed in June 2020, using the terms “cancer” with different types of cancers written out, “children and adolescents” and “high-dose methotrexate”, including also synonyms and combinations (Supplemental S2). The Cochrane Library search with the term “high-dose methotrexate” resulted in three publications, two on non-oncological diseases and one on primary central nervous system lymphoma in adults. We additionally screened the references in the LTFU care guidelines mentioned for HD-MTX and added studies fulfilling the inclusion criteria, which were not covered by the systematic search. Organs at risk, based on LTFU care guidelines, are the CNS, kidney, bone and liver. We performed this systematic review according to the guidelines of the “Preferred Reporting Items for Systematic Reviews and Meta‐Analysis” (PRISMA) [14] and registered the study protocol on PROSPERO (ID: CRD42020212262) [15].

### Inclusion and exclusion criteria

We searched for studies published in English or German between January 1985 and June 2020. Studies before 1985 were excluded as relevant treatment protocols were introduced thereafter and their focus was mainly on survival, not late effects. We defined the following inclusion criteria based on the PICO criteria: (P) study populations with ≥ 75% CCSs, defined as children and adolescents diagnosed with cancer < 19 years of age and the cancer treatment had to be completed at ≥ 12 months before late effects were assessed; (I) the cancer treatment had to contain HD-MTX, (≥ 1 g/m^2^ per application); (C) the analysis had to include either a comparison between CCSs exposed and not exposed to HD-MTX, comparison to a reference population or a regression analysis investigating the association of a certain late effects with the HD-MTX dose; (O) the outcome was not restricted on specific cancer diagnosis or organ systems at risk for late effects. We excluded case reports, case series (patient number < 10), commentaries, editorial letters, poster abstracts, narrative reviews and studies only reporting prevalence of late effects in a cohort of CCSs exposed to HD-MTX. We additionally excluded studies comparing CCSs treated with HD-MTX to CCSs treated with cranial radiotherapy with or without HD-MTX and studies reporting radiological changes in the brain as primary outcomes.

### Data extraction and quality assessment

Two reviewers (ED and KS) screened all titles and abstracts independently according to the inclusion and exclusion criteria, followed by screening the retrieved full texts for eligibility. Any disagreements were resolved by a third reviewer (MO). Data were extracted by one reviewer (ED), using a standardized data extraction form and were checked by a second reviewer (MO). The corresponding authors of seven studies were contacted to resolve uncertainties related to the data. Five authors responded and three of these studies could be included. We extracted the following information from eligible studies: (a) study details (first author, year of publication, country, study design, statistics); (b) study population (number of participants, sex, year of diagnosis or treatment, age at diagnosis, cancer type); (c) treatment characteristics (treatment protocol, HD-MTX dose per application); (d) outcome measures (method used to assess organ function, reference values, follow-up). We categorized children and adolescents receiving HD-MTX as “HD-MTX” and those without as “No HD-MTX”. If the control groups were healthy children, children diagnosed with cancer before start of treatment or patients without HD-MTX, we extracted information on their age. If any of the information was missing, we indicated this with "N/A".

Two reviewers (ED and MO) assessed the risk of bias of each eligible study independently, including attrition bias, confounding, detection bias and selection bias. A third reviewer (KS) was consulted in case of disagreement. Based on the risk of bias, an overall assessment of the available evidence was performed for each organ system ranging from very low to high quality of evidence based on the adapted GRADE criteria, used by the International Guideline Harmonization Group [16, 17].

Results were finally summarized by identified organ system at risk for late effects and synthesized descriptively.

## Results

### Literature search

**Fig. 1:**
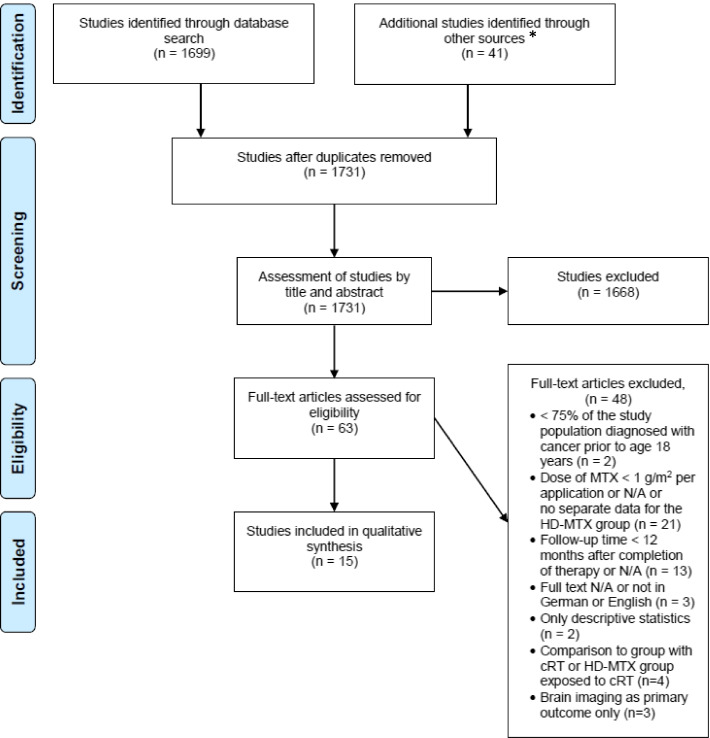
Flowchart on study selection process. Abbreviations: g/m^2^ = grams per square metre, HD-MTX = high-dose methotrexate, n = number of studies, N/A = not applicable

Through the systematic literature search, the manual search of the LTFU care guidelines and after removing duplicates, we identified 1731 studies and subsequently excluded 1668 studies after title and abstract screening. Additional 48 studies were excluded after full-text screening, mainly because the MTX dose was less than 1 g/m^2^ or the time between exposure and assessment of late affects was less than 12 months. Finally, we included 15 studies (Fig. 1) [18–32]. Ten studies assessed neurocognitive function [18–27], two kidney function [28, 29] and three bone health [30–32].

### Study populations and control groups

The size of the study populations varied between 21 and 1122 CCSs. All studies on bone health (*n* = 3) and most on neurocognitive function (*n* = 9) included CCSs diagnosed with ALL only [18–22, 24–27]. Both studies assessing renal function included different paediatric cancer types [28, 29]. The comparison groups differed between studies and consisted of childhood cancer patients with outcome assessment before start of treatment [18, 22], childhood cancer patients treated with non-HD-MTX [19–21, 31], childhood cancer patients treated with different doses of HD-MTX [27–29], healthy controls [23, 26] and reference values [21, 23–25, 27, 30, 32]. Follow-up time started in most studies at completion of treatment. The average follow-up time was below 10 years in most studies except for one study on neurocognitive function with a median follow-up of 24.7 years and one study on kidney function with a median follow-up of 15.3 years.

### Neurocognitive function

Ten studies examined the effects of HD-MTX on neurocognitive function with results from neuropsychological testing as the primary outcome [18–27] (Table 1). In all except one study [20] at least one neuropsychological test was significantly worse in CCSs treated with HD-MTX compared to the control. Different tests were carried out to assess neurocognitive function, due to different functional domains. In addition, different versions exist for some tests, such as child-, adult- and abbreviated versions of the Wechsler Intelligence Scale. This made an overall conclusion difficult. However, CCSs treated with HD-MTX performed significantly worse in at least one subtest on processing speed alone [18, 23–25]. In all studies investigating memory [23, 25] and visual-motor and fine-motor function [26], exposed CCSs performed worse than controls. In most studies on sustained attention (3 out of 4) at least one subscale was significantly lower in exposed CCSs [23–25, 27]. The same was true for memory and attention (2 out of 3 studies) [18, 21, 23], intelligence (IQ) assessment (4 out of 8 studies) [18–23, 25, 27], executive function (3 out of 6 studies) [18, 22–26], academic achievement (1 out of 3 studies) [21, 23, 27], and memory and learning (1 out of 2 studies) [22, 26]. In three neurocognitive domains, CCSs treated with HD-MTX did not perform significantly worse in any of the sub-tests than controls: attention and processing speed combined [22, 26], emotional assessment [23], and verbal learning [27] (Supplemental S3).

**Table 1:** Summary of eligible studies on late effects assessed by neuropsychological testing, *n* = 10 studies

First author Year Country	Final cohort size (male:female), diagnosis and cohort description	Age at diagnosis [years]	Follow-up [years]	Outcome variables, data type and effect of HD-MTX
Zając-Spychała et al 2017 Poland [18]	33 (17:16); ALL Study group: ▪ Group I: HD-MTX (*n*=22) *▪ Group II: HD-MTX, cRT (n=11; group not included in review)* Control group: ▪ Newly diagnosed ALL (before treatment, no CNS involvement) (*n*=12)	Group I: median 5.2 (IQR 4.3-8.2) Group II: median 4.9 (IQR 3.9-8.8)	Group I: median 4.2 (range 2.6-6.0) Group II: median 4.8 (range 2.5-6.3) Follow-up: since end of treatment	Neuropsychological testing: continuous variables Group I vs. control group: ▪ Memory and attention: 3/7 tests sign. worse in group I ▪ Processing speed: 1/2 tests sign. worse in group I ▪ Executive functions: no sign. difference in 4/4 tests ▪ IQ assessment: no sign. difference
Sherief et al 2018 Egypt [19]	100 (44:56); ALL Study group: ▪ Group I: No HD-MTX (n=44) ▪ Group II: HD-MTX (n=56)	≤ 5 (*n*=46) > 5 (*n*=54)	N/A; at least: ≥ 1 Follow-up: since end of treatment	Neuropsychological testing: continuous variables Group I vs. II: ▪ IQ assessment (full scale IQ): sign. worse in group II ▪ Verbal IQ subtests: 6/6 tests sign. worse in group II ▪ Performance IQ subtests: 4/6 tests sign. worse in group II
Halsey et al 2011 UK [20]	555 (N/A); ALL Study group: ▪ Group I (low risk): No HD-MTX (n=197) ▪ Group II (low risk): HD-MTX (n=202) ▪ *Group III (high risk):HD-MTX (n=79, not included in review as compared to group IV only)* *▪ Group IV (high risk): No HD-MTX, cRT (n=77, group not included in review)*	N/A (age at examination: median 4)	N/A; tests after 3 and 5 Follow-up: since start of therapy	Neuropsychological testing: continuous variables Group I vs. II: ▪ IQ assessment (full scale IQ): no sign. difference at 3 and 5 years ▪ Verbal IQ subtest: no sign. difference at 3 and 5 years ▪ Performance IQ subtest: no sign. difference at 3 and 5 years
Spiegler et al 2006 Canada [21]	79 (37:42); ALL Study group: ▪ Group I: HD-MTX (8.0 g/m^2^/dose) (*n*=32) ▪ Group II: VHD-MTX (33.6 g/m^2^/dose) (*n*=22) *▪ Group III: No HD-MTX, cRT (n=25, group not included in review)* Control group: ▪ Standard scores from each test	Study group: mean 2.8 ± 1.1 (range 1.0-5.0) Group I: mean 2.9 ± 1.0 (range 1.4-4.9) Group II: mean 1.9 ± 0.6 (range 1.0-3.4)	Study group: mean 10.5 ± 2.7 (range 5.1-20.6) Group I: mean 9.0 ± 1.9 (range 5.1-13.5) Group II: mean 11.8 ± 3.2 (range 5.5-20.6) Follow-up: since diagnosis	Neuropsychological testing: continuous variables Group I vs. II: ▪ Neurocognitive measures: no sign. difference in 18/18 measures in the fields of intelligence, academic achievement, attention and memory (no data shown) Group I and II (combined) vs. control group: ▪ Neurocognitive measures: 1/18 measure sign. worse in group I/II (field of attention)
Zając-Spychała et al 2018 Poland [22]	78 (46:32); ALL Study group: ▪ Group I: HD-MTX (2 g/m^2^/dose) (*n*=31) ▪ Group II: HD-MTX (5 g/m^2^/dose) (*n*=17) ▪ *Group III: HD-MTX (5 g/m*^*2*^*/dose), cRT (n=30, group not used in review)* Control group: ▪ Newly diagnosed ALL (before treatment, no CNS in-volvement; matched for age, sex) (*n*=23)	Study group: median 11.7 (IQR 1.0-9.3) Group I: median 6.2 (range 2.3-17.4) Group II: median 8.5 (range 3.3-17.0)	Median 3.8 (range 1.4- 6.3) Follow up: since end of treatment	Neuropsychological testing: continuous variables Group I vs. control group: ▪ Memory and learning: 1/4 measures sign. worse in group I ▪ Processing speed and attention: no sign. difference in 2/2 measures ▪ Executive functions: no sign. difference in 4/4 measures ▪ IQ assessment (full scale IQ): sign. worse in group I Group II vs. control group: ▪ Memory and learning: 1/4 measures sign. worse in group II ▪ Processing speed and attention: no sign. difference in 2/2 measures ▪ Executive functions: no sign. difference in 4/4 measures ▪ IQ assessment (full scale IQ): sign. worse in group II Group I vs. II: ▪ No sign. difference between the groups (trend: lower scores in processing speed, attention, visual short-term memory in group II; no data shown)
Edelmann et al 2016 US [23]	80 (46:34); Osteosarcoma Study group: ▪ Group I: HD-MTX (*n*=71; *n*=9 only patient-related outcomes) Control group: ▪ Healthy controls (matched for age, race, sex) (*n*=39) ▪ Normative population data (z-scores)	Mean 14.20	Mean 24.70 ± 6.60 Follow-up: since diagnosis	Neuropsychological and emotional testing: continuous variables Group I vs. control group: ▪ Memory: 2/3 tests sig. worse in group I ▪ Attention: 2/3 tests sig. worse in group I ▪ Processing speed: 4/4 tests sig. worse in group I ▪ Executive function: 1/3 tests sig. worse in group I ▪ Intelligence: 1/2 tests sig. worse in group I ▪ Academics: 1/2 tests sig. worse in group I ▪ Patient reported neurobehavioral functions: 2/8 tests sig. worse in group I ▪ Emotional assessment: 1/3 tests sig. worse (domain: somatization) Group I vs. population norm: ▪ Memory: 1/3 tests sig. worse in group I ▪ Attention: 2/3 tests sig. worse in group I ▪ Processing speed: 2/4 tests sig. worse in group I ▪ Executive function: 2/3 tests sig. worse in group I ▪ Intelligence: 1/2 tests sig. worse in group I ▪ Academics: 2/2 tests sig. worse in group I ▪ Patient reported neurobehavioral functions: 2/8 tests sig. worse in group I ▪ Emotional assessment: no sig. difference in 3/3 tests Higher number of HD-MTX courses, higher cumulative dose of HD-MTX, higher median peak HD-MTX concentration, higher median HD-MTX clearance, higher median HD-MTX AUC and higher cumulative HD-MTX AUC: ▪ no sign. association with abnormal neuropsychological and emotional testing (memory, attention, processing speed, executive function, reading, emotional assessment)
Liu et al 2018 US [24]	158 (76:82); ALL Study group: ▪ Group I: HD-MTX (2.5 g/m^2^/dose) (*n*=90) ▪ Group II: HD-MTX (5.0 g/m^2^/dose) (*n*=68) Control group: ▪ nrv (z-scores)	Mean 6.6 ± 4.5	Mean 7.6 ± 1.7 Follow-up: since diagnosis	Neuropsychological testing: continuous variables Group I and II (combined) vs. control group: ▪ Attention: 1/7 tests sign. worse in group I/II ▪ Processing speed: 3/7 tests sign. worse in group I/II ▪ Executive function: 4/10 tests sign. worse in group I/II Higher dose of HD-MTX (AUC): ▪ sign. association with attention problems (1/2 assessments) ▪ sign. association with processing speed problems (2/3 assessments) ▪ sign. association with executive function problems (1/5 assessments)
Fellah et al 2019 US [25]	165 (85:80); ALL Study group: ▪ Group I: HD-MTX (2.5 g/m^2^/dose) (*n*=93) ▪ Group II: HD-MTX (5.0 g/m^2^/dose) (*n*=72) Control group: ▪ nrv (z-scores)	Mean 6.7 ± 4.4	Mean 7.7 ± 1.7 Follow-up: since diagnosis	Neuropsychological testing: continuous variables Group I and II (combined) vs. control group: ▪ Memory: 1/2 tests sig. worse in group I/II ▪ Attention: no sig. difference in 4/4 tests] ▪ Processing speed: 3/7 tests sig. worse in group I/II ▪ Executive function: 4/9 tests sig. worse in group I/II ▪ Intelligence: 1/5 tests sig. worse in group I/II Higher dose of HD-MTX (AUC): ▪ sign. association with processing speed problems (no data shown) ▪ sign. association with executive function problems (1/9 tests)
Jansen et al 2008 The Netherlands [26]	49 (29:20); ALL Study group: ▪ Group I: HD-MTX (2 g/m^2^/dose) (*n*=32) ▪ Group II: HD-MTX (3 g/m^2^/dose) (*n*=17) Control group: ▪ Healthy siblings (matched for age) (n=28)	Median 6.4 (range 4.0-11.8)	Median 4.6 (range 4.1-4.9) Follow-up: since diagnosis	Neuropsychological testing: continuous variables Group I and II (combined) vs. control group: ▪ Learning and memory: no sign. difference in 3/3 assessments (no data shown) ▪ Sustained attention and speed: no sign. difference in 2/2 assessments (no data shown) ▪ Executive functioning: no sign. difference in 2/2 assessments (no data shown) ▪ Visual-motor and fine-motor function: 1/4 assessments sign. worse in group I/II, 2/4 assessments no sig. difference, 1/4 assessments no comparison done (no data shown)
Jacola et al 2016 US [27]	211 (107:104); ALL Study group: ▪ Group I: HD-MTX (2.5 g/m^2^/dose) (*n*=115) ▪ Group II: HD-MTX (5.0 g/m^2^/dose) (*n*=96) Control group: ▪ nrv	Range 1.0-18.0 < 5.0 (*n*=102) ≥ 5.0 (*n*=109)	N/A tests after 2 Follow-up: since end of treatment	Neuropsychological testing: continuous variables Group I and II (combined) vs. control group: ▪ Sustained attention: sign. more below average performance in group I/II ▪ Verbal learning: no sign. difference in 4/4 tests (no data shown) ▪ Wechsler scales: no sign. difference in 3/3 tests (no data shown) ▪ Academics: no sign. difference in 3/3 tests (no data shown) Group I vs. control group: ▪ Sustained attention: 5/5 tests sign. worse in group I ▪ Verbal learning: 1/4 tests sign. worse in group I ▪ Wechsler scales: 1/3 tests sign. worse in group I ▪ Academics (WIAT): 3/3 tests sign. worse in group I Group II vs. control group: ▪ Sustained attention: 4/5 tests sign. worse in group II ▪ Verbal learning: 1/4 tests sign. worse in group II ▪ Wechsler scales: 3/3 tests sign. worse in group II ▪ Academics: 1/3 tests sign. worse in group II Group I vs. II: ▪ Sustained attention: no sign. difference in 5/5 tests ▪ Verbal learning: no sign. difference in 4/4 tests ▪ Wechsler scales: 1/3 tests sign. worse in group II ▪ Academics: 3/3 tests sign. worse in group II Group I vs. II comparing percentage below average performance: ▪ Sustained attention: no sign. difference in 5/5 tests ▪ Verbal learning: 1/4 tests with more below average in group II; OR 0.4 (95%CI 0.2-1.0) ▪ Wechsler scales: 3/3 tests with more below average in group II Working memory: OR 0.4 (95%CI 0.2-0.9) Processing speed: OR 0.1 (95%CI 0.0-0.6) Intelligence: OR 0.3 (95%CI 0.1-0.6) ▪ Academics: 3/3 tests with more below average in group II Math: OR 0.4 (95%CI 0.2-0.8) Reading: OR 0.2 (95%CI 0.1-0.6) Spelling: OR 0.4 (95%CI 0.2-0.8) HD-MTX per 5 g/m^2^ ▪ Subscales of sustained attention, verbal learning, Wechsler Scales, academics: no sign. association of HD-MTX with adverse results

*ALL* acute lymphoblastic leukemia, *AUC* area under the curve, *CI* confidence interval, *CNS* central nervous system, *cRT* cranial radiotherapy, *HD-MTX* high-dose methotrexate, *IQR* interquartile range, *n* number, *N/A* missing information, *nrv* normal reference values, *OR* odds ratio, *sign.* significant(ly), *VHD-MTX* very high-dose methotrexate, *vs.* versus

### Kidney function

Two studies investigated kidney function in CCSs treated with HD-MTX. Mulder et al. used the glomerular filtration rate (GFR) as primary outcome [29], whereas Grönroos et al. used low-molecular weight proteinuria, albuminuria and GFR measured by isotope method [28]. All CCSs from both studies received MTX-containing treatment, but in different doses. Both studies showed no increased risk for kidney function impairment after higher cumulative doses of HD-MTX [28, 29]. Mulder et al. additionally showed no significant effect of HD-MTX on deterioration of GFR over time in their multivariable logistic regression model [29] (Table 2, Supplemental S4).

**Table 2:** Summary of eligible studies on kidney function, *n* = 2 studies

**First author** **Year** **Country**	**Final cohort (male:female), diagnosis and cohort description**	**Age at diagnosis [years]**	**Follow-up [years]**	**Method**	**Effect of HD-MTX**
Grönroos et al. 2008 Finland	28 (12:16); ALL, lymphoma	Median 7.7 (range 1.5–15.4)	Median 6.0 (range 1.0–10.0)	iGFR with ^51^CR-EDTA or ^99m^Tc-DTPA, urinalysis continuous variables	8 g/m^2^/dose vs. 5 g/m^2^/dose ▪Albuminuria: no sign. association with its occurrence (OR 1.50, 95%CI 0.29–0.78^‡^) ▪Proteinuria: no sign. association with its occurrence (OR 4.67, 95%CI 0.42–52.12)
	Study group: ▪Group I: HD-MTX (*n* = 28) 5 g/m^2^/dose: *n* = 16 8 g/m^2^/dose: *n* = 12		Follow-up: since end of treatment	Definition: ▪iGFR: reduced if iGFR ▪ < 115 ml/min/1.73m2 ▪Urinary albuminuria: abnormal if albumin/creatinine ratio > 2.5 mg/mmol	8 g/m^2^/dose vs. 5 g/m^2^/dose ▪iGFR: no sign. association with reduced iGFR
					Higher cumulative dose: ▪iGFR: no sign. association with reduced iGFR ▪Albuminuria: no sign. association with its occurrence ▪Proteinuria: no sign. association with its occurrence
Mulder et al. 2013 The Netherlands	1122 (599:523); different tumors	Median 7.6 (range 0.0–17.8)	Median 15.3 (5.0–36.1)	GFR (CKD-EPI formula for adults) continuous variables	Higher cumulative dose: ▪GFR: no sign. association with reduced GFR
	Study group: ▪Group I: HD-MTX (*n* = 253)		Follow-up: since diagnosis	Definition: ▪GFR reduced if GFR < 90 ml/min/ 1.73m^2^	Exposure: ▪GFR over time: no sign. effect of HD-MTX on deterioration of GFR over time

*ALL* acute lymphoblastic leukemia, *CI* confidence interval, *CKD-EPI* Chronic Kidney Disease Epidemiology Collaboration, *GFR* glomerular filtration rate, *HD-MTX* high-dose methotrexate, *iGFR* isotope glomerular filtration rate, *n* number, *OR* odds ratio, *sign.* significant(ly). ^‡^Numbers taken from the original article even though OR is not within the 95%CI

### Bone health

Three studies examined late effects of HD-MTX on bone health [30–32]. Bone mineral density (BMD) was assessed by dual-energy x-ray absorptiometry (DXA) (Table 3, Supplemental S5). CCSs treated with HD-MTX did not have significantly lower BMD than expected or than CCSs not treated with HD-MTX.

**Table 3:** Summary of eligible studies on bone health, *n* = 3 studies

**First author** **Year** **Country**	**Final cohort (male:female); diagnosis** **Cohort description**	**Age at diagnosis [years]**	**Follow-up [years]**	**Method**	**Effect of HD-MTX**
Lequin et al. 2002 The Nether-lands	21 (12:9); ALL	Males: mean 6.3 ± 3.5	Mean 9.6 (range 7.9–11.4)	DXA lumbar spine and total body (z-Score): BMD, BMD_vol_ continuous variables	Group I vs. control group: ▪BMD, BMD_vol_: no sign. difference
	Study group: ▪Group I: HD-MTX (*n* = 21)	Females: mean 4.6 ± 2.7	Follow-up: since end of treatment		
	Control group: ▪nrv				
Tillmann et al. 2002 UK	28 (17:11); ALL	Study group: N/A	Mean 4.5 (range 1.5–7.1)	DXA lumbar spine and total body (z-Score): BMD_vol_ continuous variables	Group I vs. II: ▪Lumbar BMD_vol_: no sign. Difference (trend: lower BMD_vol_ in group I)
	Study group: ▪Group I: HD-MTX (*n* = 18) ▪Group II: No HD-MTX (*n* = 10)	age at testing: mean 10.7 ± 2.1 (range 5.7–14.7)	Follow-up: since end of treatment		
van der Sluis et al. 2000 The Netherlands	23 (13:10); ALL	Mean 5.4 (range 1.9–12.4)	Mean 9.6 (range 7.9–11.4)	DXA lumbar spine and total body (z-Score): BMD, BMD_vol_ continuous variables	Group I vs. control group: ▪BMD, BMD_vol_: no sign. difference
	Study group: ▪Group I: HD-MTX (*n* = 23) Control group: ▪nrv		Follow-up: since end of treatment		

*ALL* acute lymphoblastic leukemia, *BMD* bone mineral density, *BMD*_*vol*_ volumetric bone mineral density, *DXA* dual-energy x-ray absorptiometry, *HD-MTX* high-dose methotrexate, *n* number, *N/A* missing information, *nrv* normal reference values, *sign.* significant(ly)

### Quality assessment

The overall quality of the evidence was low to very low for the three organ systems (Supplemental S3-S5). Following reasons led to the down-grading of the quality: selection bias, missing adjustment for important confounders and low precision, such as reporting of results as p-values only and without effect size.

## Discussion

For three organ systems, we found literature assessing the potential impact of HD-MTX on the development of late effects in CCSs: neurocognition, kidney, and bone. Based on the available literature, exposed CCSs had more often abnormal neurocognitive test results than controls but did not more frequently suffer from impaired kidney function or bone health.

For neurocognitive testing, we found evidence in most included studies (9 out of 10), that CCSs treated with HD-MTX had more frequent abnormal test results or significantly lower scores in at least one subscale compared to controls. This is congruent with the LTFU care guidelines, where all three guidelines recommend testing, either specifically for CCSs treated with HD-MTX or because it is recommended for all CCSs. Our findings are in line with a publication by Cheung et al., assessing 190 CCSs treated for ALL with chemotherapy only [33]. Even though they did not investigate MTX specifically, survivors demonstrated more neurocognitive problems in the domains of working memory, organization, initiation and planning. The pathomechanism of HD-MTX-related neurotoxicity may include alterations in intracellular metabolic pathways due to MTX-induced folate deficiency which may lead to demyelination and elevated levels of homocysteine, which can further be metabolized to excitatory neurotransmitters, causing neurotoxicity [34–36]. All but one study on neurocognitive outcomes included only CCSs treated for ALL. Intrathecal MTX is part of the standard treatment for ALL. Therefore, all patients exposed to intrathecal MTX are also exposed to HD-MTX and the potential effect of intrathecal MTX on neurocognitive function is impossible to disentangle in this setting. The only study on non-ALL survivors included 80 osteosarcoma survivors. They did not receive intrathecal MTX and performed significantly worse than the matched controls.

For kidney function, our review shows, that HD-MTX does not have a significantly negative impact in the long-term [28, 29]. This is reflected in the LFTU care guidelines, where it is recommended in one guideline only (Supplemental S1). It is unclear how HD-MTX contributes to kidney function impairment years following completion of treatment. For acute nephrotoxicity the mechanism includes precipitation of MTX and its metabolites within the renal tubules [37]. The methods used to assess kidney function were urinalysis, radioisotop GFR and calculation of GFR using the Chronic Kidney Disease Epidemiology Collaboration (CKD-EPI) formula for adults. Urinalysis and estimated GFR (eGFR) based on serum creatinine level are widely used in daily practice. Although the eGFR is standardized for age, sex and ethnic origin, it might under- or overestimate the true GFR, as the creatinine is influenced by the muscle mass. This is the case, if a patients muscle mass deviates from the mean of the reference population of the same sex and age [38]. Cystatin C varies less with anthropometric data. It has been shown that cystatin C-based GFR formulas can provide an accurate estimation of GFR in the paediatric population, including oncology patients [39, 40]. We therefore suggest using cystatin C or cystatin C-based GFR alone or in addition to the eGFR to estimate kidney function in CCSs.

Bone mineral density in CCSs treated with HD-MTX does not significantly differ from the reference values. This contrasts with some LTFU care guidelines, where it either is recommended or not specified (Supplemental S1). This finding is also unexpected, as all three studies included ALL survivors only. Survivors treated for ALL are exposed to high-dose steroids, which are associated with a reduction in BMD [41]. HD-MTX may cause reduced bone mass by several mechanisms, including growth plate dysfunction, damage to osteoprogenitor cells resulting in suppressed bone formation, and increase bone resorption [42]. Our results might be explained by the small cohorts and the missing information on physical activity, nutrition intake and other hormonal deficits in the included studies, which are relevant determinants for BMD [31, 43].

### Strengths and limitations

This is the first review summarizing late effects of HD-MTX treatment on different organ systems in CCSs. The key strengths of this study are the thorough application of the systematic review methodology, including the performance of all steps by two reviewers, and the quality assessment of the included studies. The neurocognitive domains assessed, the tests used for each domain and the reference values differed between studies; for example in the Wechsler test [19–21]. This made the direct comparison of results from the different studies difficult. Most recently, guidelines and position statements on screening strategies and neurocognitive testing have been published [44, 45]. Similar guidelines would be helpful to assess kidney and bone health in a standardized way. Because of the small numbers of eligible studies and their small population sizes, the results on kidney and bone health might not be representative for all CCSs exposed to HD-MTX. Furthermore, important confounders were not considered, for example socioeconomic status in neuropsychological testing or physical activity for bone health. In addition, the overall quality of the evidence was low to very low.

## Conclusion

CCSs treated with HD-MTX are at risk for neurocognitive impairment, but not for significantly worse kidney function or bone health than controls. These findings are in contrast to the currently used LTFU care guidelines, where also kidney, bone, and liver are defined as organs at risk. Further research is needed to fully understand the impact of HD-MTX on late effects in CCSs and its relevance for long-term follow-up care.

## Supplementary Information

**Additional file 1:Supplemental S1.** Recommendation for late effects screening in different long-term follow-up care guidelines. **Supplemental S2.** Search strategy in PubMed. **Supplemental S3.** Detailed summary of eligible studies on late effects assessed by neuropsychological testing, *n* = 10 studies. **Supplemental S4.** Detailed summary of eligible studies on kidney function, *n* = 2 studies. **Supplemental S5.** Detailed summary of eligible studies on bone health, *n* = 3 studies.

## Data Availability

The search strategy is available in the supplemental material as additional material.

## Abbreviations

ALL: Acute Lymphoblastic Leukemia
BMD: Bone Mineral Density
CCS: Childhood Cancer Survivor
CKD-EPI: Chronic Kidney Disease Epidemiology Collaboration
CNS: Central Nervous System
COG: Children’s Oncology Group
DXA: Dual-energy X-ray Absorptiometry
DCOG: Dutch Childhood Oncology Group
eGFR: Estimated Glomerular Filtration Rate
GFR: Glomerular Filtration Rate
HD-MTX: High-dose Methotrexate
LTFU: Long-term follow-up
MTX: Methotrexate
UKCCLG: United Kingdom Children’s Cancer Study Group Late Effects Group
