# Supplementary material for: Late effects of high-dose methotrexate treatment in childhood cancer survivors—a systematic review
Source: BMC Cancer. 2022 Mar 14;22:267. doi: 10.1186/s12885-021-09145-0 (PMC8919635; doi:10.1186/s12885-021-09145-0)
Supplement: Supplementary file 1 — Additional file 1:Supplemental S1. Recommendation for late effects screening in different long-term follow-up care guidelines. Supplemental S2. Search strategy in PubMed. Supplemental S3. Detailed summary of eligible studies on late effects assessed by neuropsychological testing, n = 10 studies. Supplemental S4. Detailed summary of eligible studies on kidney function, n = 2 studies. Supplemental S5. Detailed summary of eligible studies on bone health, n = 3 studies. [file 12885_2021_9145_MOESM1_ESM.docx]

**Late effects of high-dose methotrexate treatment in childhood cancer survivors - a systematic review**

Journal of Cancer Survivorship

Eveline Daetwyler, Mario Bargetzi, Maria Otth, Katrin Scheinemann

**Corresponding author:**

Maria Otth, MD

Division of Hematology-Oncology, Department of Pediatrics

Kantonsspital Aarau AG

Tellstrasse 25

CH-5001 Aarau

Switzerland

Mail: maria.otth@ksa.ch

**Supplemental material**

| **LTFU care guideline** | **Neuropsychological** | **Kidney** | **Bone** | **Liver** |
| --- | --- | --- | --- | --- |
| **COG (5)** | recommended | not recommended | recommended | recommended |
| **UKCCLG (6)** | recommended for all CCSs | recommended | recommendation unclear | recommended |
| **DCOG (7)** | recommended for all CCSs | not included | not included | recommended |

**Supplemental S1**: Recommendation for late effects screening in different long-term follow-up care guidelines

Abbreviations: COG, Children’s Oncology Group; DCOG, the Dutch Childhood Oncology Group; LTFU, long-term follow-up; UKCCLG, United Kingdom Children’s Cancer Study Group Late Effects Group;

**Supplemental S2:** Search strategy in PubMed

| #1 | **"Neoplasms"[MesH] OR "Bone Marrow Transplantation"[MesH]** |
| --- | --- |
| #2 | **leukemi*[tiab] OR leukaemi*[tiab] OR AML[tiab] OR lymphom*[tiab] OR hodgkin*[tiab] OR non-hodgkin[tiab] OR sarcom*[tiab] OR Ewing*[tiab] OR germ cell*[tiab] OR astrocytoma*[tiab] OR craniopharyngeoma[tiab] OR osteosarcom*[tiab] OR wilms*[tiab] OR nephroblastom*[tiab] OR neuroblastom*[tiab] OR rhabdomyosarcom*[tiab] OR teratom*[tiab] OR hepatom*[tiab] OR hepatoblastom*[tiab] OR medulloblastom*[tiab] OR PNET*[tiab] OR neuroectodermal tumor*[tiab] OR retinoblastom*[tiab] OR meningiom*[tiab] OR gliom*[tiab] OR brain tumor*[tiab] OR brain tumour*[tiab] OR brain neoplasm*[tiab] OR central nervous system neoplasm*[tiab] OR central nervous system tumor*[tiab] OR central nervous system tumour*[tiab] OR brain cancer*[tiab] OR intracranial neoplasm*[tiab] OR testis neoplasm[tiab] OR testicular neoplasm*[tiab] OR testis cancer*[tiab] OR testicular cancer*[tiab] OR testis tumor*[tiab] OR testis tumour*[tiab] OR cancer of testis[tiab] OR testis neoplasm*[tiab] OR oncol*[tiab] OR cancer*[tiab] OR tumor[tiab] OR tumors[tiab] OR tumour*[tiab] OR tumours[tiab] OR neoplas*[tiab] OR malignanc*[tiab] OR (transplantation[tiab] AND bone marrow[tiab]) OR hematopoietic stem cell[tiab] OR haematopoietic[tiab] OR stem cell[tiab]** |
| #3 | **"Child"[MesH] OR "Infant"[MesH] OR "Adolescent"[MesH] OR "Pediatrics"[MesH]** |
| #4 | **child*[tiab] OR infan*[tiab] OR adolescen*[tiab] OR newborn*[tiab] OR new-born*[tiab] OR baby*[tiab] OR babies[tiab] OR neonat*[tiab] OR perinat*[tiab] OR postnat*[tiab] OR schoolchild*[tiab] OR school child*[tiab] OR kid[tiab] OR kids[tiab] OR toddler*[tiab] OR teen*[tiab] OR boy*[tiab] OR girl*[tiab] OR juvenil*[tiab] OR youth*[tiab] OR young*[tiab] OR kindergar*[tiab] OR pediatric*[tiab] OR paediatric*[tiab] OR school*[tiab] OR preschool*[tiab] OR pre school*[tiab] OR elementary school*[tiab] OR highschool*[tiab] OR high school*[tiab] OR schoolage*[tiab] OR school age*[tiab]** |
| #5 | **#1 OR #2** |
| #6 | **#3 OR #4** |
| #7 | **#5 AND #6** |
| #8 | **"Animals"[Mesh] NOT "Humans"[Mesh]** |
| #9 | **#7 NOT #8** |
| #10 | **("high-dose"[Title/Abstract] OR "highdose"[Title/Abstract]) OR "high dose"[Title/Abstract]) AND ("methotrexate"[Title/Abstract] OR "methotrexate"[MeSH Terms])** |
| #11 | **#9 AND #10** |

**Supplemental S3:** Detailed summary of eligible studies on late effects assessed by neuropsychological testing, n = 10 studies

| First author  Year  Country | Study design | Final cohort  (male:female)  Diagnosis / treatment year  Cohort description  References | Age at diagnosis  [years] | Diagnosis | Treatment protocol | MTX dose per application  [g/m^2^] | Follow-up  [years] | Method | Effect of HD-MTX | Risk of bias |
| --- | --- | --- | --- | --- | --- | --- | --- | --- | --- | --- |
| O. Zając-Spychała  2017  Poland | Unicenter, prospective, cross-sectional | 33 [61]  (17:16) 2002-2007  Study group:   - Group I: HD-MTX (n=22) - *Group II: HD-MTX, cRT (n=11); group not included in review*   Control group:   - Newly diagnosed ALL (before treatment, no CNS involvement) (n=12)   Reference for neuropsycho-logical testing: Polish standardized version | Group I:  median 5.2 (IQR 4.3-8.2)  Group II:  median 4.9 (IQR 3.9-8.8)  Control group: median 11.8 (IQR 9.2-13.7) | ALL | ALL IC-BFM 2002 protocol | 2 g/m^2^  or  5 g/m^2^ | Group I: median 4.2 (range 2.6-6.0)  Group II: median 4.8 (range 2.5-6.3)  Follow-up: since end of treatment | Neuro-psychological testing (Wechsler Intelligence Test for Children-revised, Rey Auditory Verbal Learning Test, Verbal Fluency Test, Benton Visual Retention Test, Stroop Test, Clock Drawing Test, Wisconsin Card Sorting Test)  continuous variables | Statistical analysis:   - Statistical significance level at p < 0.05 - Mann-Whitney U test - p-values only   Results:  Group I vs. control:   - Memory and attention: 3/7 tests sign. worse in group I - Processing speed: 1/2 tests sign. worse in group I - Executive functions: no sign. difference in 4/4 tests - IQ assessment: no sign. difference | SB: high risk  AB: low risk  DB: unclear  CF: high risk |
| L.M. Sherief  2018  Egypt | Multicenter,  prospective, cross-sectional | 100 [N/A]  (44:56) N/A  Study group:   - Group I: No HD-MTX (n=44) - Group II: HD-MTX (n=56)   Reference for neuropsycho-logical testing: Arabic standardized version | ≤ 5 (n=46)  > 5 (n=54) | ALL | Modified CCG 1991 protocol (Group I), St. Jude Total XV protocol (Group II) | 2.5 g/m^2^  or  5 g/m^2^ | N/A  at least: ≥ 1  Follow-up: since end of treatment | Neuro-psychological testing (Arabic version of Wechsler Intelligence Test for children-III)  continuous variables | Statistical analysis:   - Statistical significance level at p ≤ 0.05 - Student’s t-test - p-values only   Results:  Group I vs. II   - IQ assessment (full scale IQ): sign. worse in group II - Verbal IQ subtests: 6/6 tests sign. worse in group II - Performance IQ subtests: 4/6 tests sign. worse in group II | SB: high risk  AB: low risk  DB: unclear  CF: high risk |
| Chr. Halsey  2011  UK | Multicenter, prospective, cross-sectional | 555 [1826]  (N/A) 1990-1997  Study group:   - Group I (low risk): No HD-MTX (n=197) - Group II (low risk): HD-MTX (n=202) - *Group III (high risk): HD-MTX (n=79); not included in review as compared to group IV only* - *Group IV (high risk): No HD-MTX, cRT (n=77); group not included in review*   Reference for neuropsycho-logical testing: test standardization norms | N/A (age at testing: median 4) | ALL | UKALL XI protocol | 6 g/m^2^ (3 courses)  or  8 g/m^2^ (3 courses) | N/A  tests after 3 and 5  Follow-up: since start of therapy | Neuro-psychological testing (Wechsler Intelligence Test for preschool and primary scale-revised/for children-III/for adults-revised)  continuous variables | Statistical analysis:   - Statistical significance level at p ≤ 0.02 - Student’s t-test - Diff. in means with 95%CI   Results:  Group I vs. II:   - IQ assessment (full scale IQ): no sign. difference. at 3 and 5 years - Verbal IQ subtest: no sign. difference at 3 and 5 years - Performance IQ subtest: no sign. difference at 3 and 5 years | SB: high risk  AB: low risk  DB: unclear  CF: low risk |
| B.J. Spiegler  2006  Canada | Unicenter, retrospective,  cross-sectional | 79 [120] (37:42) 1983-1996  Study group:   - Group I: HD-MTX  (8.0 g/m^2^/dose) (n=32) - Group II: VHD-MTX (33.6 g/m^2^/dose) (n=22) - *Group III: No HD-MTX, cRT (n=25), group not included in review*   Control group:   - Standard scores from each test   Calcualtion of age-adjusted z-scores based on standard scores of each standardized test | Study group:  mean 2.8 ± 1.1 (range 1.0-5.0)  Group I:  mean 2.9 ± 1.0 (range 1.4-4.9)  Group II:  mean 1.9 ± 0.6 (range 1.0-3.4) | ALL | Adapted BFM study group | 8.0 g/m^2^ (3 courses)  or  33.6 g/m^2^ (3 courses) | Study group:  mean 10.5  ± 2.7 (range 5.1-20.6)  Group I:  mean 9.0  ± 1.9 (range 5.1-13.5)  Group II: mean 11.8  ± 3.2 (range 5.5-20.6)  Follow-up: since diagnosis | Neuro-psychological testing (Wechsler Intelligence Test for children-III/for adults-III, Wide Range Achievement Test 3^rd^ edition, Woodcock Reading Mastery Test-Revised, Gordon Diagnostic System, Children’s Memory Scale, Wechsler Memory Scale-III)  continuous variables | Statistical analysis:   - Statistical significance level at p ≤ 0.003 - One sample t-test, multivariate linear regression - p-values only   Results:  Group I vs. II:   - Neurocognitive measures: no sign. difference in 18/18 measures in the fields of intelligence, academic achievement, attention and memory (no data shown)   Group I and II (combined) vs. control:   - Neurocognitive measures: 1/18 tests sign. worse in group I/II (field of attention) | SB: high risk  AB: low risk  DB: unclear  CF: low risk |
| O. Zając-Spychała  2018  Poland | Unicenter, prospective, cross-sectional | 78 [166]  (46:32) 2002-2012  Study group:   - Group I: HD-MTX  (2 g/m^2^/dose) (n=31) - Group II: HD-MTX (5 g/m^2^/dose) (n=17) - *Group III: HD-MTX  (5 g/m^2^/dose),* cRT (n=30); *group not used in review*   Control group:   - Newly diagnosed ALL (before treatment, no CNS involvement; matched for age, sex) (n=23)   Reference for neuropsycho-logical testing: Polish standardized version | Study group:  median 11.7 (IQR 1.0-9.3)  Group I:  median 6.2 (range 2.3-17.4)  Group II:  median 8.5 (range 3.3-17.0)  Control group:  median 11.4 (IQR 9.3-13.1) | ALL | ALL IC-BFM 2002,  ALL IC-BFM 2009 | 2 g/m^2^  or  5 g/m^2^ | Median 3.8 (range 1.4- 6.3)  Follow up: since end of treatment | Neuro-psychological testing (Wechsler Intelligence Test for Children, Rey Auditory Verbal Learning Test, Verbal Fluency Test, Stroop Test, Processing Speed Index of Wechsler Scale, selective attention of Stroop Test Performance Index, Clock Drawing Test, Wisconsin Card Sorting Test)  continuous variables | Statistical analysis:   - Statistical significance level at p < 0.05 - Student’s t test, ANOVA - p-values only   Results:  Group I vs. control:   - Memory and learning: 1/4 measures sign. worse in group I - Processing speed and attention: no sign. difference in 2/2 measures - Executive functions: no sign. difference in 4/4 measures - IQ assessment (full scale IQ): sign. worse in group I   Group II vs. control:   - Memory and learning: 1/4 measures sign. worse in group II - Processing speed and attention: no sign. difference in 2/2 measures - Executive functions: no sign. difference in 4/4 measures - IQ assessment (full scale IQ): sign. worse in group II   Group I vs. II:   - No sign. difference Between the groups  (trend: lower scores in processing speed, attention, visual short-term memory in group II; no data shown) | SB: high risk  AB: low risk  DB: unclear  CF: low risk |
| M.N. Edelmann  2016  US | Unicenter, prospective, cross-sectional | 80 [120]  (46:34) 2008-2014  Study group:   - Group I: HD-MTX (n=71; n=9 only patient-related outcomes)   Control group:   - Healthy controls (matched for age, race, sex) (n=39) - Normative population data (z-scores)   Reference for neuropsycho-logical testing: test standardization norms | Study group:  mean 14.20  Control group (age at testing):  mean 39.03  ± 11.71 | Osteo-sarcoma | St. Jude Osteosarcoma protocol 72, 77, 86, 91;  Multi-Institutional Osteo-sarcoma protocol (MIOS) | 2.5 g/m^2^  or  5 g/m^2^  or  12 g/m^2^ | Mean 24.70 ± 6.60  Follow-up:  since diagnosis | Neuro-psychological testing (Wechsler Abbreviated Scale of Intelligence/ Wechsler Adult Intelligence Scale-III, Woodcock-Johnson-III Tests of Achievement, Trail Making Test Part A/B, Conner’s Continuous Performance Test [CPT]-II, California Verbal Learning Test-II, CPT-II hit rate, the Grooved Pegboard Test, Controlled Oral Word Association Test, Behavior Rating Inventory of Executive Function),  emotional assessment (Brief Symptom Inventory-18, Short Form 36)  continuous variables | Statistical analysis:   - Statistical significance level at p ≤ 0.01 - Two group t-test / one sample t-test, multivariate analysis - p-values and 95%CI   Results:  Group I vs. control:   - Memory: 2/3 tests sign. worse in group I - Attention: 2/3 tests sign. worse in group I - Processing speed: 4/4 tests sign. worse in group I - Executive function: 1/3 tests sign. worse in group I - Intelligence: 1/2 tests sign. worse in group I - Academics: 1/2 tests sign. worse in group I - Patient reported neurobehavioral functions: 2/8 tests sign. worse in group I - Emotional assessment: 1/3 tests sign. worse (domain: somatization)   Group I vs. population norm:   - Memory: 1/3 tests sign. worse in group I - Attention: 2/3 tests sign. worse in group I - Processing speed: 2/4 tests sign. worse in group I - Executive function: 2/3 tests sign. worse in group I - Intelligence: 1/2 tests sign. worse in group I - Academics: 2/2 tests sign. worse in group I - Patient reported neurobehavioral functions: 2/8 tests sign. worse in group I - Emotional assessment: no sign. difference in 3/3 tests   HD-MTX:   - No sig. association of the number of HD-MTX courses, cumulative dose of HD-MTX, median peak HD-MTX concentration, median HD-MTX clearance, median HD-MTX AUC and cumulative HD-MTX AUC with neuropsychological and emotional testing (memory, attention, processing speed, executive function, reading, emotional assessment; multivariate analysis) | SB: high risk  AB: low risk  DB: unclear  CF: low risk |
| W.Liu  2018  US | Unicenter, prospective, cross-sectional | 158 [220]  (76:82) 2000-2010  Study group:   - Group I: HD-MTX (2.5 g/m^2^/dose) (n=90) - Group II: HD-MTX (5.0 g/m^2^/dose) (n=68)   Control group:   - nrv (z-scores)   Reference for neuropsycho-logical testing: test standardization norms | Mean 6.6  ± 4.5 | ALL | St. Jude Total Therapy Study XV protocol | 2.5 g/m^2^ (4 courses)  or  5.0 g/m^2^ (4 courses) | Mean 7.6  ± 1.7  Follow-up: since diagnosis | Neuro-psychological testing (Conners’ Continuous Performance Test CPT II, Conners’ Parent Rating Scale CPRS, Delis-Kaplan Executive Function System, Wechsler Intelligence Test for Children, Rey Complex Figure Copy, The Grooved Pegboard Test)  continuous variables | Statistical analysis:   - Statistical significance level at p < 0.05 - Two sample t-test, multiple linear regression   Results:  Group I and II (combined) vs. control:   - Attention: 1/7 tests sign. worse in group I/II - Processing speed: 3/7 tests sign. worse in group I/II - Executive function: 4/10 tests sign. worse in group I/II   HD-MTX:   - Sign. association of higher dose of HD-MTX (AUC) with attention problems (1/2 assessments), processing speed problems (2/3 assessments), executive function problems (1/5 assessments) (general linear models, adjustment for age at diagnosis, parents’ education) | SB: high risk  AB: low risk  DB: unclear  CF: low risk |
| S.Fellah  2019  US | Unicenter, prospective, cross-sectional | 165 [302]  (85:80) 2000-2010  Study group:   - Group I: HD-MTX (2.5 g/m^2^/dose) (n=93) - Group II: HD-MTX (5.0 g/m^2^/dose) (n=72)   Control group:   - nrv (z-scores)   Reference for neuropsycho-logical testing: Wefel et al. (2011) | Mean 6.7  ± 4.4 | ALL | St. Jude Total Therapy Study XV protocol | 2.5 g/m^2^ (4 courses)  or  5.0 g/m^2^ (4 courses) | Mean 7.7  ± 1.7  Follow-up:  since diagnosis | Neuro-psychological assessement (Number Letter Switch, Verbal Fluency from the Delis-Kaplan Executive Function System, Digit Backward Test from Wechsler Scale, Continuous Performance Test Omissions / Variability / Detectability Indices, Attention Network Task, tests for Intelligence, Memory, Processing Speed)  continuous variables | Statistical analysis:   - Statistical significance level at p < 0.05 - One sample t-test, Pearson’s test - Repeated measures, analysis of variance   Results:  Group I and II (combined) vs. control:   - Memory: 1/2 tests sign. worse in group I/II - Attention: no sign. difference in 4/4 tests - Processing speed: 3/7 tests sign. worse in group I/II - Executive function: 4/9 tests sign. worse in group I/II - Intelligence: 1/5 tests sign. worse in group I/II   HD-MTX:   - Sign. association of higher HD-MTX exposure (AUC) with processing speed problems (no data shown) and executive function problems (1/9 tests, flexibility) (multivariate analysis) | SB: high risk  AB: low risk  DB: unclear  CF: high risk |
| N. Jansen  2008  The Netherlands | Unicenter, prospective, longitudinal | 49 [N/A]  (29:20) N/A  Study group:   - Group I: HD-MTX  (2 g/m^2^/dose) (n=32) - Group II: HD-MTX (3 g/m^2^/dose) (n=17)   Control group:   - Healthy siblings (matched for age) (n=28)   Reference for neuropsycho-logical testing: Dutch standardized version | Study group: median 6.4 (range 4.0-11.8)  Control group (age at testing): median 8.2 (range 4.5-12.6) | ALL | ALL-9 protocol of the Dutch Childhood Leukemia Study Group | 2 g/m^2^ (3 courses)  or  3 g/m^2^ (4 courses) | Median 4.6 (range 4.1-4.9)  Follow-up: since diagnosis | Neuro-psychological testing (Dutch version of Rey’s Auditory Verbal Learning Test: Learning and Recall; Rey-Osterrieth Complex Figure Test: Delayed Recall and Copy; Bourdon-Vos Self-paced Continuous-Performance Cancellation Task: Speed and Accuracy; Wisconsin Card Sorting Test: Errors and Perseverations; Beery Developmental Test of Visual-Motor Integration; Purdue Pegboard: Simple Motor Functioning and Higher-Order Motor Functioning)  continuous variables | Statistical analysis:   - Statistical significance level at p < 0.05 - Multivariate multilevel models - p-values only   Results:  Group I and II (combined) vs. control:   - Learning and memory: no sign. difference in 3/3 assessments (no results shown) - Sustained attention and speed: no sign. difference in 2/2 assessments (no results shown) - Executive functioning: no sign. difference in 2/2 assessments (no results shown) - Visual-motor and fine-motor functioning:  1/4 assessments sign. worse in group I/II, 2/4 assessments no sig. difference, 1/4 assessments no comparison done (no results shown) | SB: high risk  AB: low risk  DB: unclear  CF: high risk |
| L.M. Jacola 2016 US | Unicenter, prospective, longitudinal | 211 [408]  (107:104) 2000-2007  Study group:   - Group I: HD-MTX  (2.5 g/m^2^/dose) (n=115) - Group II: HD-MTX (5.0 g/m^2^/dose) (n=96)   Control group:   - nrv   Reference for neuropsycho-logical testing: test standardization norms | Range 1.0-18.0  < 5.0 (n=102)  ≥ 5.0 (n=109) | ALL | St. Jude Total Therapy Study XV protocol | 2.5 g/m^2^ (4 courses)  or  5.0 g/m^2^ (4 courses) | N/A  tests after 2  Follow-up: since end of treatment | Neuro-psychological testing (Bayley Scales of Infant Development 2^nd^ edition, Continuous Performance Test: Attentiveness, Hit Reaction Time, Variability, Risk-taking, Omissions; California Verbal Learning Test: List A Total, Learning Slope, Short Delay Free Recall, Long Delay Free Recall; Wechsler Scales: Working Memory, Processing Speed; Academics: Math, Reading, Spelling)  continuous variables | Statistical analysis:   - Statistical significance level at p ≤ 0.05 - One sample t-test, Fischer exact (x^2^) test, multivariable regression analysis   Results:  Group I and II (combined) vs. control:   - Sustained attention: sign. higher frequency of below average performance in group I/II - Verbal learning: no sign. difference in 4/4 tests (no results shown) - Wechsler scales: no sign. difference in 3/3 tests (no results shown) - Academics: no sign. difference in 3/3 tests (no results shown)   Group I vs. control:   - Sustained attention: 5/5 tests sign. worse in group I - Verbal learning: 1/4 tests sign. worse in group I - Wechsler scales: 1/3 tests sign. worse in group I - Academics (WIAT): 3/3 tests sign. worse in group I   Group II vs. control:   - Sustained attention: 4/5 tests sign. worse in group II - Verbal learning: 1/4 tests sign. worse in group II - Wechsler scales: 3/3 tests sign. worse in group II - Academics: 1/3 tests sign. worse in group II   Group I vs. II:   - Sustained attention: no sign. difference in 5/5 tests - Verbal learning: no sign. difference in 4/4 tests - Wechsler scales: 1/3 tests sign. worse in group II - Academics: 3/3 tests sign. worse in group II   Group I vs. II comparing percentage below average performance:   - Sustained attention: no sign. difference in 5/5 tests - Verbal learning: 1/4 tests with higher frequency of below average in group II: OR 0.4 (95%CI 0.2-1.0) - Wechsler scales: 3/3 tests with higher frequency of below average in group II  Working memory: OR 0.4 (95%CI 0.2-0.9) Processing speed: OR 0.1 (95%CI 0.0-0.6)   Intelligence: OR 0.3 (95%CI 0.1-0.6)   - Academics: 3/3 tests with higher frequency of below average in group II Math: OR 0.4 (95%CI 0.2-0.8) Reading: OR 0.2 (95%CI 0.1-0.6) Spelling: OR 0.4 (95%CI 0.2-0.8)   HD-MTX   - HD-MTX per 5 g/m^2^: no sig. correlations in subscales of sustained attention, Verbal learning, Wechsler Scales, Academics (multivariable logistic regression analysis) | SB: high risk  AB: low risk  DB: unclear  CF: low risk |
| **GRADE assessment**:  Study design: +4 Prospective cohort studies  Study limitations: -1 Limitations: Selection bias high in 10/10; Attrition bias low in 10/10, Detection bias low in 10/10; Confounding bias high in 4/10  Consistency: 0 No important inconsistency (in 9/10 studies: HD-MTX with adverse effect on neuropsychological testing)  Directness: 0 Results are direct, population and outcomes broadly generalizable  Precision: -1 Mostly p-values, no effect size  Publication bias: 0 Unlikely  Effect size: 0 No large magnitude of effect  Dose-response: 0 No dose response relationship  Plausible confounding: 0 No plausible confounding | | | | | | | | | | |
| **Quality of evidence:** ⊕⊕⊖⊖ low quality evidence  **Conclusion:** Consistent findings with 9 of 10 studies revealing adverse outcomes in at least one neuropsychological sub-test performed in childhood cancer survivors exposed to HD-MTX compared to non-exposed or reference values. | | | | | | | | | | |
| **Abbreviations**: AB = attrition bias, CF = confounding, DB = detection bias, SB = selection bias; ALL = acute lymphoblastic leukemia, AUC = area under the curve, CI = confidence interval, CNS = central nervous system, cRT = cranial radiotherapy, HD-MTX = high-dose methotrexate, IQR = interquartile range, n = number, N/A = missing information, nrv = normal reference values, OR = odds ratio, SD = standard deviation, VHD-MTX = very high-dose methotrexate, vs. = versus | | | | | | | | | | |

**Supplemental S4**: Detailed summary of eligible studies on kidney function, n = 2 studies

| First author  Year  Country | Study design | Final cohort  (male:female)  Diagnosis / treatment year  Cohort description  References | Age at diagnosis [years] | Diagnosis | Treatment protocol | MTX dose per application  [g/m^2^] *) | Follow-up  [years] | Method | Effect of HD-MTX | Risk of bias |
| --- | --- | --- | --- | --- | --- | --- | --- | --- | --- | --- |
| M.H. Grönroos  2008  Finland | Multicenter, prospective and retro-spective, longitudinal | 28 [N/A]  (12:16) 1992-2003  Study group:   - Group I: HD-MTX (n=28)   Reference for GRF: Schwartz et al., 2007; Reference for blood pressure: Soergel et al., 1997 | Median 7.7 (range 1.5-15.4) | ALL (n=25), lymphoma (n=3) | Modified COMP-DAUNO-protocol, Nordic Society of Pediatric Haemato-logy and Oncology (NOPHO) protocol) | 5 g/m^2^ (2-9 courses) (n=16)  or  8 g/m^2^ (2-9 courses) (n=12) | Median 6.0 (range 1.0-10.0)  Follow-up: since end of treatment | iGFR with ^51^CR-EDTA or ^99m^Tc-DTPA, urinalysis,  continuous variables  Definition:   - iGFR: reduced if iGFR < 115 ml/min/1.73m2 - Urinary albuminuria: abnormal if albumin / creatinine ratio >  2.5 mg/mmol | Statistical analysis:   - Statistical significance level at p < 0.05 - Univariable regression analysis   Results:   - No sign. association of higher cumulative. dose or dose per application  (8 versus 5 g/m^2^/dose) with occurrence of albuminuria (OR 1.50, 95%CI 0.29-0.78^‡^)) - No sign. association of cumulative dose or dose per application (8 versus 5 g/m^2^/dose) with occurrence of proteinuria (OR 4.67, 95%CI 0.42-52.12) - No sign. association of higher cumulative dose or dose per application (8 versus 5 g/m^2^/dose) with reduced iGFR | SB: high risk  AB: low risk  DB: low risk  CF: high risk |
| R.L. Mulder  2013  The Nether-lands | Unicenter, prospective, longitudinal | 1122 [1251]  (599:523) 1966-2003  Study group:   - Group I: HD-MTX (n=253)   Reference for GRF: National Kidney Foundation, 2002 | Median 7.6 (range 0.0-17.8) | Leukemia (n=267), lymphoma (n=259), renal (n=144),  STS (n=125), bone tumor (n=99), brain (n=77), NB (n=57),  GCT (n=45), RB (n=11), hepatic (n=10), others (n=28) | N/A | HD-MTX defined as  > 1.0 g/m^2^/ dose  Cumulative dose:  median 22.5 (range 1.5-138.0) | Median 15.3 (5.0-36.1)  Follow-up:  since diagnosis | GFR (CKD-EPI formula for adults)  continuous variables  Definition:   - GFR reduced if GFR < 90ml/min/ 1.73m^2^ | Statistical analysis:   - Statistical significance level at p < 0.01 - Multivariable linear random effects models - p-values only   Results:   - No sign. association of higher cumulative dose of HD-MTX with reduced GFR - No sign. effect of HD-MTX exposure on deterioration of GFR over time | SB: low risk  AB: low risk  DB: low risk  CF: low risk |
| **GRADE assessment**:  Study design: +4 Prospective cohort studies  Study limitations: -2 Limitations: Selection bias high in 1/2; Attrition bias low in 2/2; Detection bias low in 2/2; Confounding high in 1/2  Consistency: 0 Consistent results  Directness: 0 Results are direct, population and outcomes broadly generalizable  Precision: -1 Only 2 studies, 1/2 studies with multivariable analysis  Publication bias: 0 Unlikely  Effect size: 0 No large magnitude of effect  Dose-response: 0 No dose response relationship  Plausible confounding: 0 No plausible confounding | | | | | | | | | | |
| **Quality of evidence:** ⊕⊖⊖⊖ very low quality evidence  **Conclusion:** Both studies showed no increased risk for impaired kidney function after higher cumulative doses of HD-MTX, measured by GFR, iGFR, proteinuria or albuminuria. | | | | | | | | | | |
| **Abbreviations:** AB = attrition bias, CF = confounding, DB = detection bias, SB = selection bias; ALL = acute lymphoblastic leukemia, CI = confidence interval, CKD-EPI = Chronic Kidney Disease Epidemiology Collaboration, GCT = germ cell tumour, GFR = glomerular filtration rate, HD-MTX = high-dose methotrexate, iGFR = isotope glomerular filtration rate, n = number, N/A = missing information, NB = neuroblastoma, OR = odds ratio, RB = retinoblastoma, sign. = significant(ly), STS = soft tissue sarcoma | | | | | | | | | | |
| **Remark:** *) MTX defined as dose per application if not otherwise specified  ^‡^) Numbers taken from the original article even though OR is not within the 95%CI | | | | | | | | | | |

**Supplemental S5:** Detailed summary of eligible studies on bone health, n = 3 studies

| First author  Year  Country | Study design | Final cohort  (male:female)  Diagnosis / treatment year  Cohort description  References | Age at diagnosis [years] | Diagnosis | Treatment protocol | MTX dose per application  [g/m^2^] | Follow-up  [years] | Method | Effect of HD-MTX | Risk of bias |
| --- | --- | --- | --- | --- | --- | --- | --- | --- | --- | --- |
| M.H. Lequin  2002  The Nether-lands | Unicenter, prospective, cross-sectional | 21 [N/A]  (12:9) N/A  Study group:   - Group I: HD-MTX (n=21)   Control group:   - nrv (Boot et al., 1997) | Males:  mean 6.3 ± 3.5   Females:  mean 4.6 ± 2.7 | ALL | ALL-6 protocol of the Dutch Childhood Leukemia Study Group | 2 g/m^2^ (4 courses) | Mean 9.6 (range 7.9-11.4)  Follow-up: since end of treatment | DXA lumbar spine and total body  (z-Score): BMD, BMD_vol_  continuous variables | Statistical analysis:   - Statistical significance level at p ≤ 0.05 - One sample t-test - p-values only   Results:  Group I vs. control:   - BMD, BMD_vol_: no sign. difference | SB: high risk  AB: low risk  DB: low risk  CF: high risk |
| V. Tillmann  2002  UK | Unicenter, prospective,  cross-sectional | 28 [31]  (17:11) N/A  Study group:   - Group I: HD-MTX (n=18) - Group II: No HD-MTX (n=10) | Study group: N/A  age at testing: mean 10.7 ± 2.1 (range 5.7-14.7)  Control group (age at testing): mean 10.4 ± 3.1 (range 5.2-15.8) | ALL | UKALL XI protocol | 6 g/m^2^ (3 courses)  or 8 g/m^2^ (3 courses) | Mean 4.5 (range 1.5-7.1)  Follow-up: since end of treatment | DXA lumbar spine and total body  (z-Score): BMC, BMD_vol_, BA, BA for height, areal BMD  continuous variables | Statistical analysis:   - Statistical significance level at p ≤ 0.05 - Student’s t-test - p-values only   Results:  Group I vs. II:   - Lumbar BMD_vol_: no sig. diff. (trend: lower BMD_vol_ in group I) - BMC, BA, BA for height, areal BMD: no results shown | SB: low risk  AB: low risk  DB: low risk  CF: high risk |
| I.M.van der Sluis  2000  The Nether-lands | Unicenter, prospective, cross-sectional | 23 [N/A]  (13:10) 1984-1988  Study group:   - Group I: HD-MTX (n=23)   Control group:   - nrv (Boot et al., 1997) | Mean 5.4 (range 1.9-12.4) | ALL | ALL-6 protocol of the Dutch Childhood Leukemia Study Group | 2 g/m^2^ (3 courses)  Mean: 8.2 | Mean 9.6 (range 7.9-11.4)  Follow-up:  since end of treatment | DXA lumbar spine and total body  (z-Score): BMD, BMD_vol_  continuous variables | Statistical analysis:   - Statistical significance level at p < 0.05 - One sample t-tests - p-values only   Results:  Group I vs. control:   - BMD, BMD_vol_: no sign. difference | SB: high risk  AB: low risk  DB: low risk  CF: high risk |
| **GRADE assessment**:  Study design: +4 Prospective cohort studies  Study limitations: -2 Limitations: Selection bias high in 2/3; Attrition bias low in 3/3; Detection bias low in 3/3; Confounding bias high in 3/3  Consistency: 0 No inconsistency (in 3/3 studies: HD-MTX showed no effect on bone health)  Directness: 0 Results are direct, population and outcomes broadly generalizable  Precision: -1 Mostly p-values only, no effect size (3/3 studies)  Publication bias: 0 Unlikely  Effect size: 0 No large magnitude of effect  Dose-response: 0 No dose response relationship  Plausible confounding: 0 No plausible confounding | | | | | | | | | | |
| **Quality of evidence:** ⊕⊖⊖⊖ very low quality evidence  **Conclusion:** Consistent finding with no significant negative effect of HD-MTX on bone mineral density. | | | | | | | | | | |
| **Abbreviations:** AB = attrition bias, CF = confounding, DB = detection bias, SB = selection bias; ALL = acute lymphoblastic leukemia, BA = bone area, BMC = bone mineral content, BMD = bone mineral density, BMD_vol_ = volumetric bone mineral density, DXA = dual-energy x-ray absorptiometry, HD-MTX = high-dose methotrexate, n = number, N/A = missing information, nrv = normal reference values, QCT = quantitative computed tomography, sig. = significant(ly), UKALL = United Kingdom Acute Lymphoblastic Leukaemia, | | | | | | | | | | |
